# Prediction of single-cell mechanisms for disease progression in hypertrophic remodelling by a trans-omics approach

**DOI:** 10.1038/s41598-021-86821-y

**Published:** 2021-04-14

**Authors:** Momoko Hamano, Seitaro Nomura, Midori Iida, Issei Komuro, Yoshihiro Yamanishi

**Affiliations:** 1 Department of Bioscience and Bioinformatics, Faculty of Computer Science and Systems Engineering, Kyushu Institute of Technology, 680-4 Kawazu, Iizuka, Fukuoka 820-8502 Japan; 2 Department of Cardiovascular Medicine, Graduate School of Medicine, The University of Tokyo, Tokyo, 113-8655 Japan; 3 Genome Science Division, Research Center for Advanced Science and Technologies, The University of Tokyo, Tokyo, 153-0041 Japan

**Keywords:** Computational biology and bioinformatics, Cardiology

## Abstract

Heart failure is a heterogeneous disease with multiple risk factors and various pathophysiological types, which makes it difficult to understand the molecular mechanisms involved. In this study, we proposed a trans-omics approach for predicting molecular pathological mechanisms of heart failure and identifying marker genes to distinguish heterogeneous phenotypes, by integrating multiple omics data including single-cell RNA-seq, ChIP-seq, and gene interactome data. We detected a significant increase in the expression level of natriuretic peptide A (*Nppa*), after stress loading with transverse aortic constriction (TAC), and showed that cardiomyocytes with high *Nppa* expression displayed specific gene expression patterns. Multiple NADH ubiquinone complex family, which are associated with the mitochondrial electron transport system, were negatively correlated with *Nppa* expression during the early stages of cardiac hypertrophy. Large-scale ChIP-seq data analysis showed that Nkx2-5 and Gtf2b were transcription factors characteristic of high-*Nppa-*expressing cardiomyocytes. *Nppa* expression levels may, therefore, represent a useful diagnostic marker for heart failure.

## Introduction

Heart failure is one of the most serious problems associated with cardiovascular diseases, as reflected by an increase in the number of affected patients^1^. Cardiac hypertrophy develops in response to hemodynamic overload to maintain cardiac function; however, this adaptive process results in cardiac dysfunction over time, manifesting as heart failure^2,3^. Heart failure is a heterogeneous disease with multiple risk factors and various pathophysiological types, which makes it difficult to identify and understand the molecular mechanisms involved^4,5^.

An emerging approach for exploring the heterogeneity of diseases is single-cell RNA-sequencing (RNA-seq), which has recently been applied to medical research on various diseases. Recent studies reported that cardiomyocytes demonstrate heterogeneous molecular mechanisms of heart failure. For example, the activation of mitochondrial translation/metabolism-related genes are correlated with cell size and linked to extracellular signal-related kinase ERK1/2 and nuclear respiratory factor NRF1/2 transcriptional networks during the process of heart failure^6^. Cardiac hypertrophy has also been reported to occur with spatial and temporal heterogeneity in myosin heavy chain β (*Myh7*) expression in cardiomyocytes after pressure overload^7^. These studies suggested that cardiomyocyte gene expression at the single-cell level, can influence cardiac phenotypes and therefore cardiac functions. Elucidating the molecular mechanisms of heart failure by revealing conserved gene expression programs related to cardiac function at the single-cell level is expected to improve the accurate assessment and prediction of medical treatment responses of cardiomyocytes and progression of cardiac pathology.

The identification of the molecular signatures that underlie the heterogeneity of heart failure is a challenging issue faced by precision medicine. Molecular signatures play important roles in the choice of optimal treatment regimes, based on a patient's genetic or biochemical background. For example, blood biochemical B-natriuretic peptide (BNP) and N-terminal (NT)-proBNP have been widely used as biomarkers of heart failure for diagnosis and prognosis^8,9^, however, it is very difficult to characterize the heterogeneity of heart failure using existing biomarkers. Histology can more accurately assess the variety of pathophysiological processes compared with blood biochemical examination, and facilitating the identification of spatial heterogeneity within the heart^10^. The analysis of large-scale single-cell omics data for heart failure is expected to reveal the molecular pathological mechanisms associated with the heterogeneity at the single-cell level. The data-driven extraction of molecular signatures that may explain the heterogeneity of heart failure is, therefore, required.

In this study, we proposed a trans-omics approach for predicting molecular mechanisms for disease progression in heart failure at the single cell level, and identifying marker genes that explain the heterogeneity of heart failure, by integrating multiple omics data including single-cell RNA-seq, chromatin immunoprecipitation sequencing (ChIP-seq), and gene interactome data. Most biological functions are involved in various molecular interaction networks such as metabolic pathways, signalling pathways, and gene regulatory networks. Biological phenomena can be observed at multiple omics layers, including transcriptome, regulome, and interactome. Thus, we attempted to perform an integrative analysis of heart failure-related transcriptome data combined with other omics data in a trans-omics framework.

## Results

### Overview of the proposed trans-omics approach

**Figure 1:**
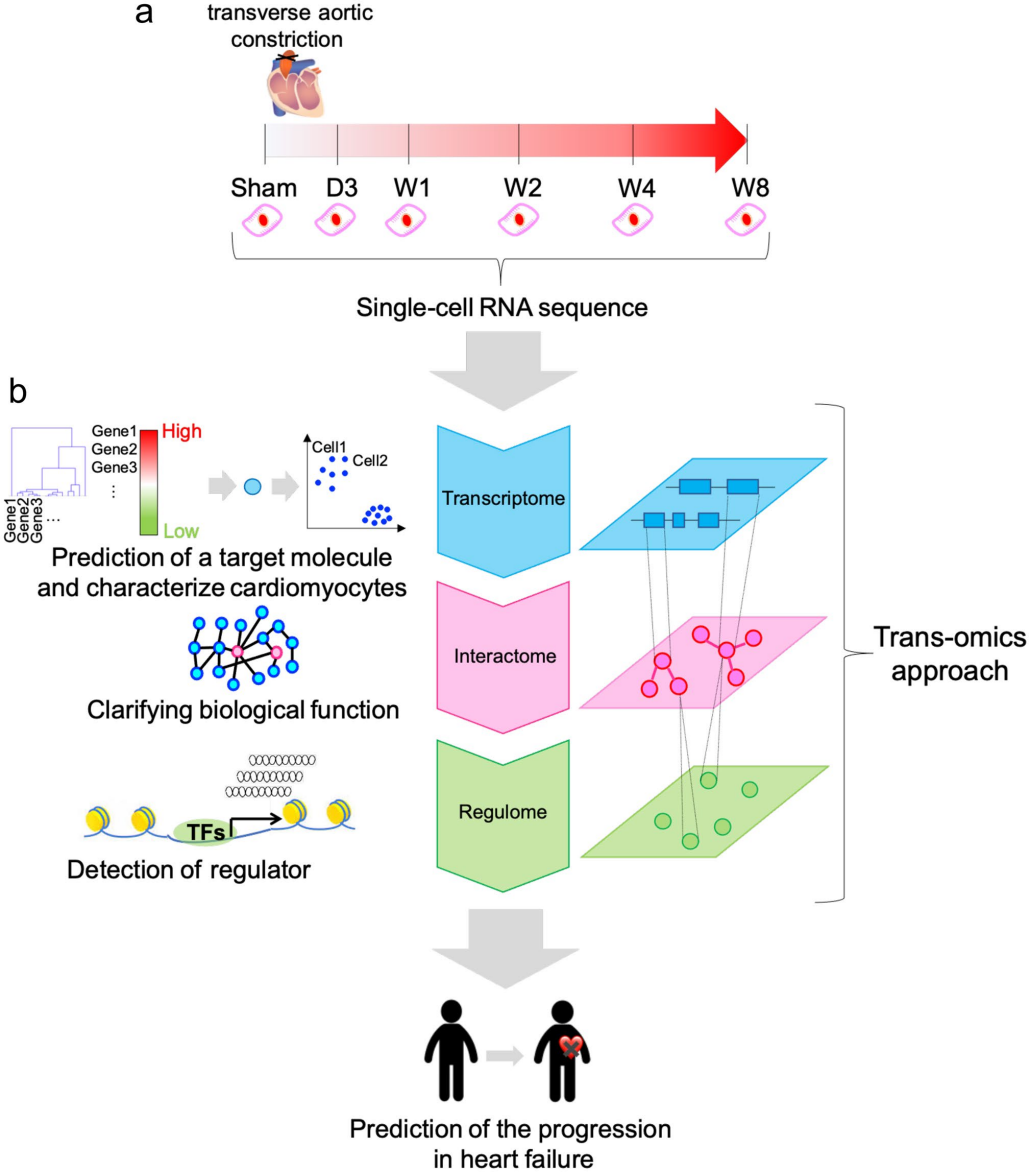
Overview of the analysis of single-cardiomyocyte transcriptome data by a trans-omics approach. (**a**) Single-cell RNA-seq data were acquired from mice exposed to pressure overload by transverse aortic constriction (TAC) or sham operation. Day 3 (D3), week 1 (W1), week 2 (W2), week 4 (W4) and week 8 (W8). Marker genes were identified by time-series gene expression profiles at the single-cell level. (**b**) Biological functions of the marker genes are estimated by a network analysis of interactome data, and regulators of the marker genes are detected by a large-scale analysis of ChIP-seq data.

Figure 1 shows an overview of our proposed trans-omics approach. First, we acquired single-cardiomyocyte RNA-seq data, from both mice that underwent transverse aortic constriction (TAC) and control mice that underwent a sham operation, using cardiomyocytes collected 3 days and 1, 2, 4 and 8 weeks after TAC, to identify disease-related genes, which we called marker genes, based on a time-series analysis of the transcriptome data at the single-cell level (Fig. 1a). Next, we estimated the central biological functions of cardiomyocytes characterized by marker genes, using network analysis of the interactome data. Finally, we detected the transcription factors characteristic of those cardiomyocytes characterized by marker genes by performing large-scale analysis of publicly available ChIP-seq data, reflecting the regulome of transcription factors (Fig. 1b). The detailed procedures can be found in the Methods section.

### Detection of genes with expression change in a single cardiomyocyte in the process of heart failure

To detect the genes with changes across the time points for the sham group and at 3 days and 1, 2, 4 and 8 weeks after TAC, we performed analysis of variance (ANOVA) on time-series RNA-seq data. This analysis detected 3135 genes with significant change of expression level among the time points.

**Figure 2:**
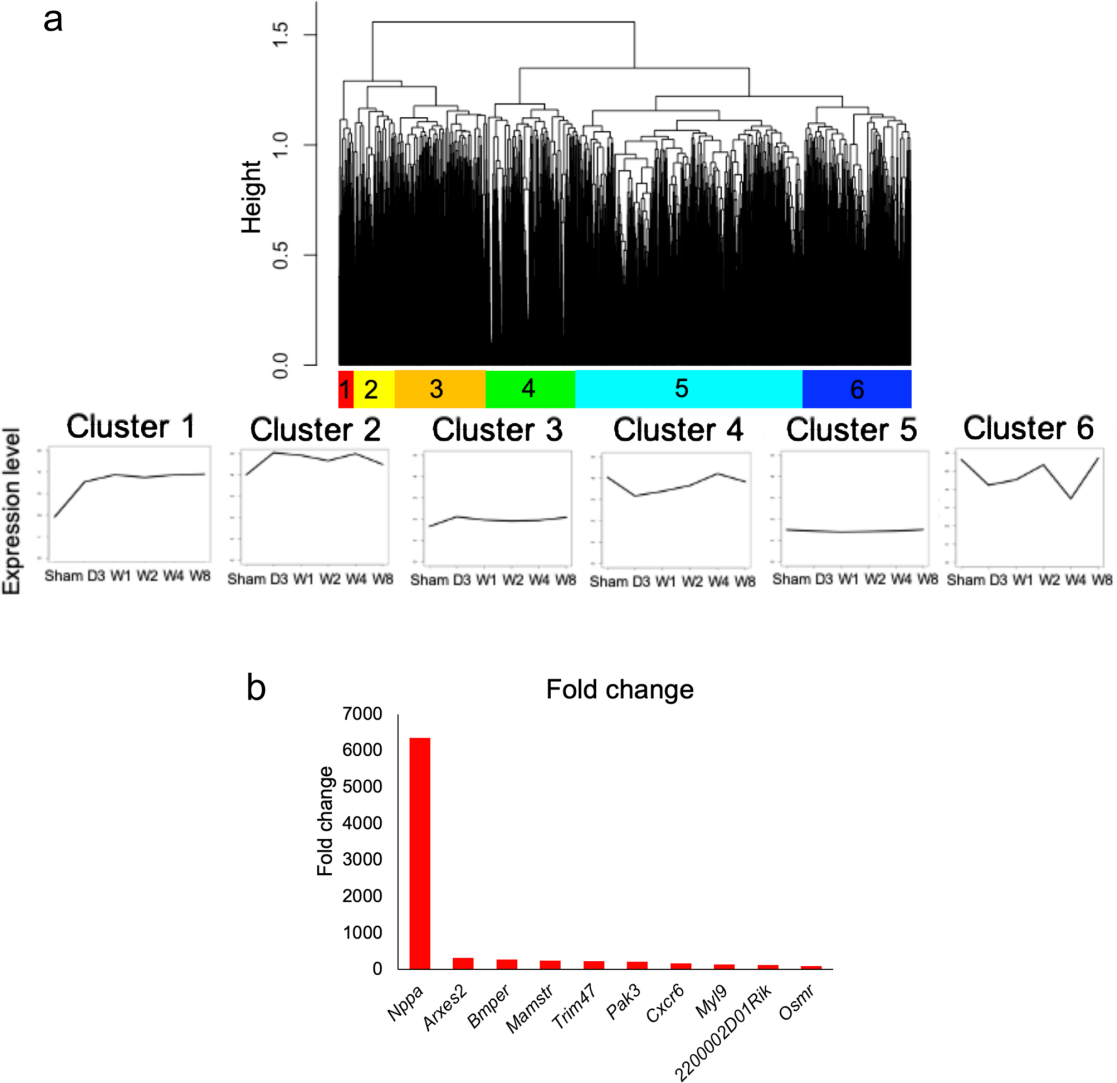
Identification of genes with expression change in single cardiomyocytes in the process of heart failure. (**a**) The top panel shows a dendrogram of hierarchical clustering of genes with a temporal change of expression identified by ANOVA. Each of the bottom panels shows the average gene expression levels in each of the six clusters. The horizontal axis of each panel indicates the time points (sham, D3, W1, W2, W4, W8) and the vertical axis indicates the average of gene expression levels. (**b**) Bar plot of top 10 genes with the greatest fold change among the upregulated genes in cluster 1.

Next, we performed hierarchical clustering on the 3135 genes with expression change, and identified six different clusters with distinct expression patterns. Figure 2a shows a dendrogram of the hierarchical clustering of gene expression patterns for the 3135 genes, and the average gene expression pattern in each of six clusters. For the cluster 1 genes, the average expression level was promptly upregulated at 3 days after TAC and maintained at a high level compared to sham until 8 weeks. Thus, we focused on cluster 1 genes because they rapidly responded to stress and were suspected of being associated with heart failure deterioration^11,12^. To examine the biological functions of all genes in cluster 1, we additionally performed an enrichment analysis using Gene Ontology (GO) terms. The list of the associated GO terms of the genes in cluster 1 is shown in Supplementary Table 1.

To identify the most highly upregulated genes, we calculated the fold change of gene expression of cluster 1 genes against that in the sham group at day 3 after TAC. Figure 2b shows the top 10 genes in cluster 1 in terms of the largest fold expression change. Among the cluster 1 genes, fold change of the *Nppa* expression level was the highest, at 6355-fold, among the genes at day 3 after TAC. Therefore, we focused on *Nppa* and investigated its expression pattern in detail.

### *Nppa* expression level varied among cardiomyocytes after TAC

**Figure 3:**
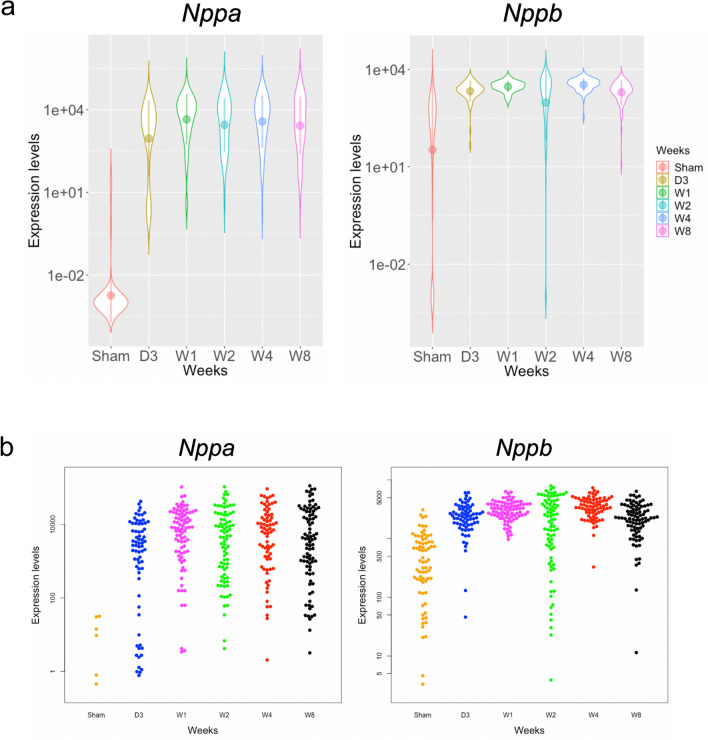
*Nppa* and *Nppb* expression levels in single cardiomyocytes and non-cardiomyocytes after TAC. (**a**) Violin plot on the left shows the distribution of *Nppa* expression levels at each time point. Violin plot on the right shows the distribution of *Nppb* expression levels at each time point. (**b**) Dot plot on the left shows the distribution of *Nppa* expression levels at each time point. Dot plot on the right shows the distribution of *Nppb* expression levels at each time point.

We examined the *Nppa* upregulation at the single cell level and compared it with the expression of *Nppb*, its paralog^13^. Figure 3a shows violin plots for the distributions of the *Nppa* and *Nppb* expression levels at time points. Figure 3b shows dot plots for the distributions of the *Nppa* and *Nppb* expression levels at time points. From these figures, it was observed that that the *Nppa* expression level was upregulated at day 3 and then maintained at a high level until 8 weeks. Notably, the *Nppa* expression level varied among cardiomyocytes at each time point, while the *Nppb* expression level did not. This result suggests that the upregulation of *Nppa* exhibits cell-to-cell heterogeneity after TAC.

### Cardiomyocytes with high *Nppa* expression had specific gene expression patterns compared with sham-group cardiomyocytes

**Figure 4:**
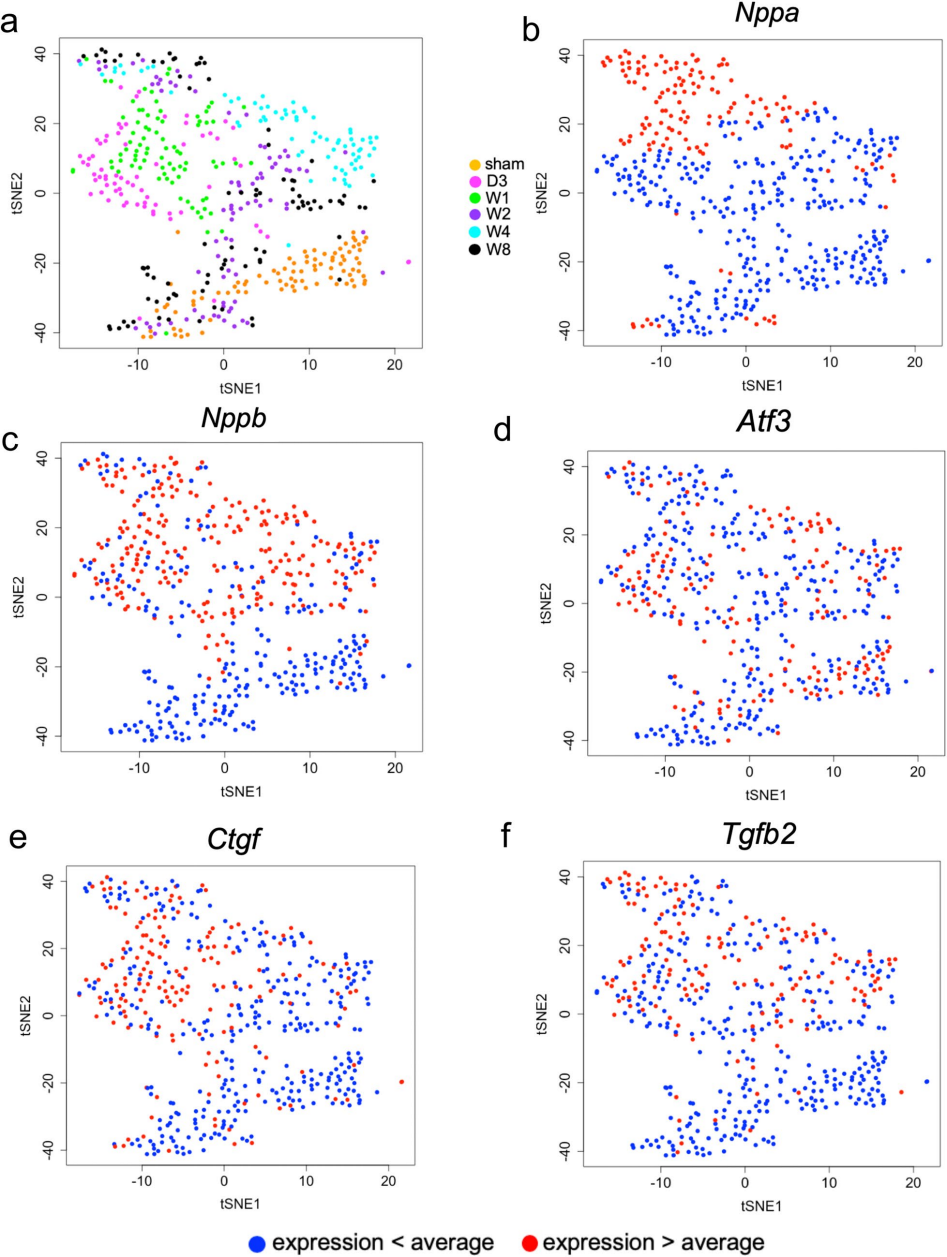
Distribution of cell-to-cell transcriptional variation in single cardiomyocytes. (**a**) Scatter-plot shows t-distributed stochastic neighbour embedding (t-SNE) visualization of cardiomyocytes. Cells represented by dots are coloured to reflect the time points when they were extracted. (**b–f**) Scatter-plots show t-SNE visualization of cardiomyocytes. Cells represented by dots are coloured to reflect the expression levels of *Nppa* (**b**), *Nppb* (**c**), *Atf3* (**d**), *Ctgf* (**e**) and *Tgfb2* (**f**). Red dots indicate cardiomyocytes in which the expression levels of *Nppa* (**b**), *Nppb* (**c**), *Atf3* (**d**), *Ctgf* (**e**) and *Tgfb2* (**f**) were higher than average, while blue dots indicate those in which the expression levels of *Nppa* (**b**), *Nppb* (**c**), *Atf3* (**d**), *Ctgf* (**e**) and *Tgfb2* (**f**) were lower than the average.

To visualize cell-to-cell variations, we performed t-distributed stochastic neighbour embedding (t-SNE) for dimension reduction of gene expression profiles. Figure 4a shows a plot of the t-SNE coordinates for all cardiomyocytes, where colors represent sham-operation and TAC-treatment at different time points. Sham-operated cardiomyocytes were densely clustered together. In contrast, TAC-treated cardiomyocytes were dispersed regardless of time point. The results suggest that sham-operated cardiomyocytes have similar gene expression patterns, while TAC-treated cardiomyocytes have high variability in gene expression patterns and in a time-independent manner.

We next investigated the gene expression patterns of cardiomyocytes with high expression of *Nppa* versus low *Nppa* expression, in the tSNE map. Figure 4b shows the distribution of cardiomyocytes based on the expression level of *Nppa*. One hundred forty-two cardiomyocytes with high expression of *Nppa* were separately clustered from those with low expression. Cardiomyocytes with high expression of *Nppa* (colored in red in Fig. 3b) were located furthest away from sham-operated cardiomyocytes (colored in orange in Fig. 4a). Moreover, cardiomyocytes with high *Nppa* expression were densely distributed, whereas those with high *Nppb* expression were not, as shown in Fig. 4c. These results suggest that cardiomyocytes with high *Nppa* expression have similar gene expression patterns and they have different gene expression patterns from sham-operated cardiomyocytes.

Several molecules have been proposed as cardiac stress response markers, so we compared the distributions of cardiomyocytes with high expression of *Nppa* with those of other stress response markers such as *Atf3* (Fig. 4d)^14^, *Ctgf* (Fig. 4e)^15^ and *Tgfb2* (Fig. 4f)^16^. Unlike *Nppa*, cardiomyocytes with high expression of *Atf3*, *Ctgf* and *Tgfb2* were not densely distributed. These results suggest that only cardiomyocytes with high *Nppa* expression exhibit similar gene expression patterns, in contrast to sham-operated cardiomyocytes.

### Cardiomyocytes with high and low *Nppa* expression differed in terms of differentially expressed genes and their biological functions

To understand the differences between cardiomyocytes with high and low *Nppa* expression, we divided cardiomyocytes into two subgroups by taking the average *Nppa* expression level as a threshold. The numbers of cardiomyocytes with high *Nppa* expression in which *Nppa* expression was higher than average on day 3 and weeks 1, 2, 4 and 8 were 19, 36, 27, 28 and 32, respectively, while the corresponding numbers of cardiomyocytes with low *Nppa* expression in which *Nppa* expression was lower than the average were 50, 47, 55, 45 and 55, respectively (Supplementary Fig. 1).

**Figure 5:**
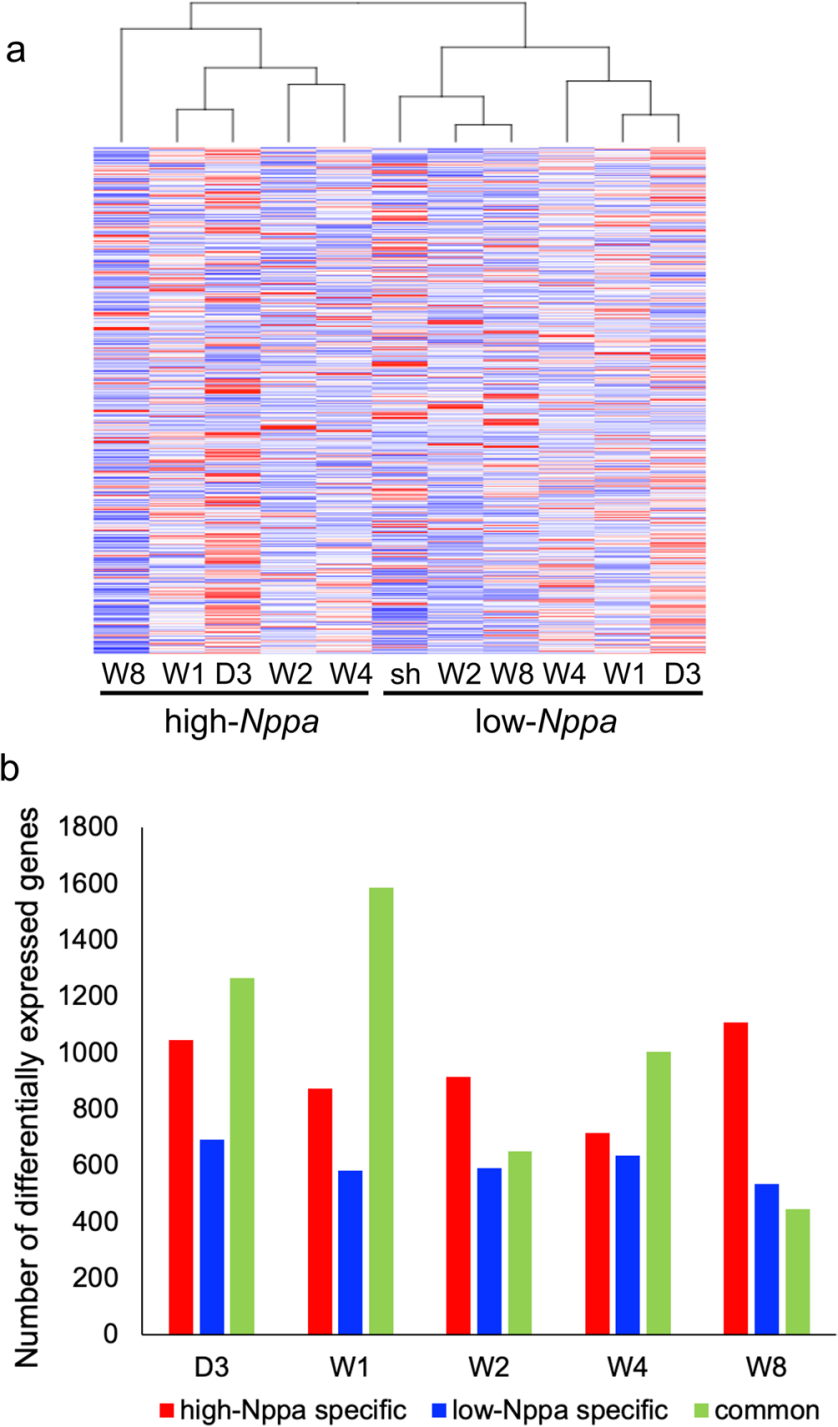
The difference of gene expression patterns in cardiomyocytes with high and low *Nppa* expression. (**a**) Hierarchical clustering of both genes and samples based on their gene expression profiles in the gene expression matrix. Cardiomyocytes were divided into two subgroups based on *Nppa* expression level; high-*Nppa* group and low-*Nppa* group. The average gene expression levels were calculated for samples from each time point in the high-*Nppa* group and low-*Nppa* group. (**b**) Bar graph shows the number of DEGs detected in high-*Nppa*, low-*Nppa* and common group, compared with sham group. “Common genes” indicate DEGs present in both high-*Nppa* expressing cardiomyocytes and low-*Nppa* expressing cardiomyocytes.

For both subgroups, we calculated the average gene expression level of cardiomyocytes at each time point, and then performed hierarchical clustering based on the average gene expression levels over time. Figure 5a shows a dendrogram of the hierarchical clustering of the subgroups at different time points. It was observed that cardiomyocyte subgroups with high and low *Nppa* expression were clustered furthest apart, implying that cardiomyocytes with high and low *Nppa* expression differed in terms of gene expression patterns.

We then compared TAC-induced and sham-operated cardiomyocytes to identify differentially expressed genes (DEGs) by applying unpaired two-tailed Student’s t-test with |log_2_Fold Change|≥ 1. Figure 5b shows the resulting numbers of DEGs in cardiomyocytes with high and low *Nppa* expression and those of common genes in cardiomyocytes with both high and low *Nppa* expression at each time point. “Common genes” indicate DEGs present in both high-*Nppa* expressing cardiomyocytes and low-*Nppa* expressing cardiomyocytes. It was observed that DEGs in cardiomyocytes with high *Nppa* expression were largely different from those in cardiomyocytes with low *Nppa* expression.

We further investigated the differences in biological functions of DEGs between cardiomyocytes with high and low *Nppa* expression by performing gene set enrichment analysis (GSEA). Supplementary Table 1 shows the enriched Gene Ontology (GO) categories associated with genes among high-*Nppa*-expressing cardiomyocytes compared with those with low expression. A variety of GO terms for biological functions were detected in *Nppa* high-expressing cardiomyocytes compared to *Nppa* low-expressing cardiomyocytes. For example, we detected the GO terms “Regulation of transcription elongation from RNA polymerase II promoter’ and ‘DNA packing’ at day 3 after TAC, implying that transcriptional regulation is promptly activated in cardiomyocytes with high *Nppa* expression. These results suggest that cardiomyocytes with high and low *Nppa* expression are different in terms of biological functions of DEGs.

### *Nppa* upregulation correlates with Hdac2 induction

**Figure 6:**
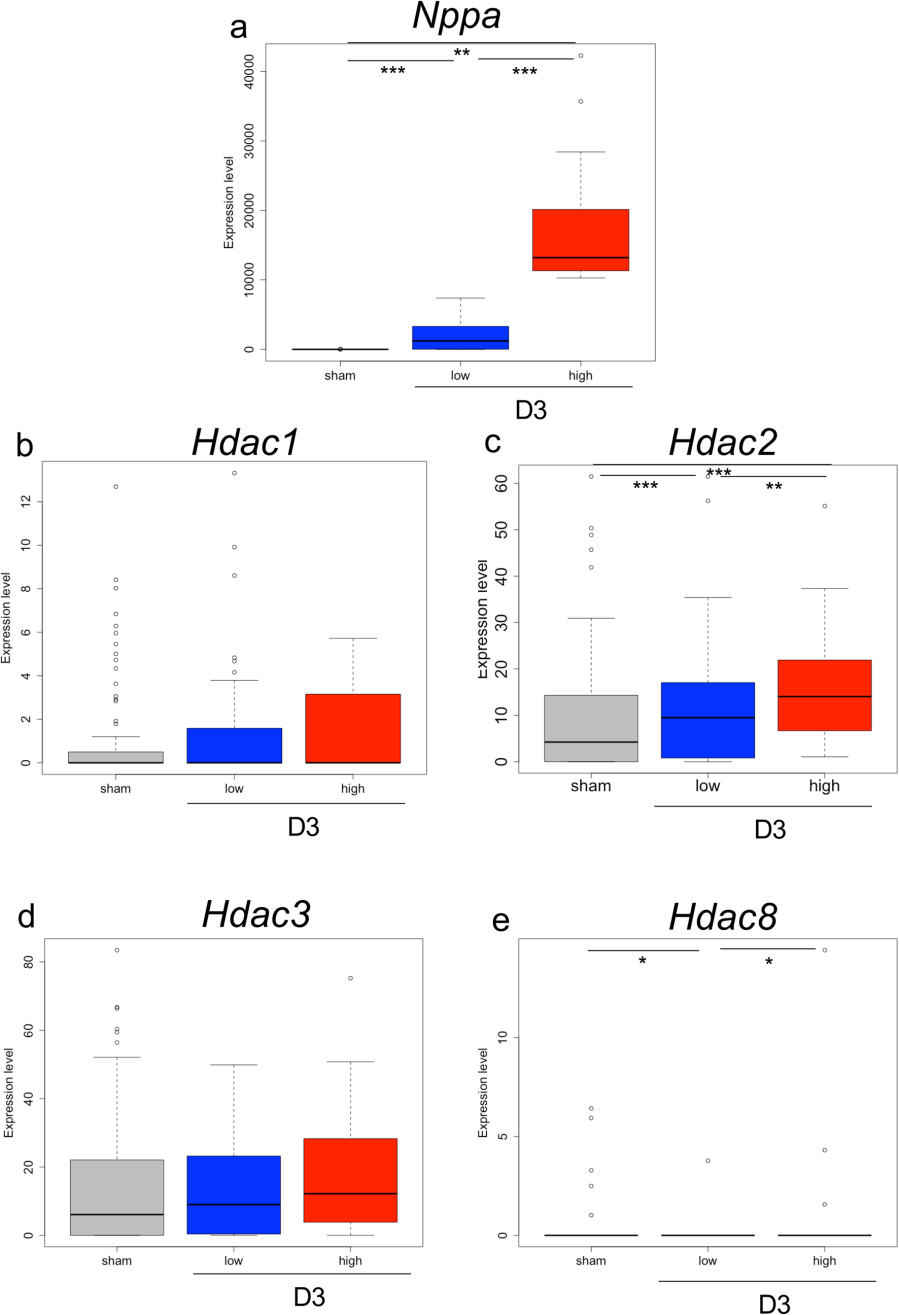
*Nppa* expression and Hdac class I expression in cardiomyocytes. (**a–e**) Box plots show the expression levels of *Nppa* (**a**), *Hdac1* (**b**), *Hdac2* (**c**), *Hdac3* (**d**) and *Hdac8* (**e**) at day 3 after TAC. Blue columns show the expression levels in cardiomyocytes with low *Nppa* expression and red columns show the expression levels in cardiomyocytes with high *Nppa* expression.

We examined the pathway upstream of *Nppa* induction at day 3 after TAC. It was reported that *Nppa* transcription was upregulated in the heart by histone deacetylase (Hdac) 2 activity under loading stress^17^. To confirm the activity of Hdac2 at day 3 after TAC at the single-cell level, we investigated the expression levels of the following Hdac class I members: *Hdac1*, *Hdac2*, *Hdac3* and *Hdac8*. Figure 6 shows the distributions of expression levels of *Nppa* and Hdac class I members in cardiomyocytes. It was observed that the expression level of *Hdac2* was significantly upregulated in *Nppa* high-expressing groups compared with that in low-expressing group. On the other hand, the expression levels of *Hdac1*, *Hdac3* and were not significantly upregulated. The expression of *Hdac8* was not observed in most cardiomyocytes. These results suggest that the initial response to TAC of *Nppa* induction is regulated by Hdac2 at the single-cell level.

### Biological functions of cardiomyocytes with high *Nppa* expression

**Figure 7:**
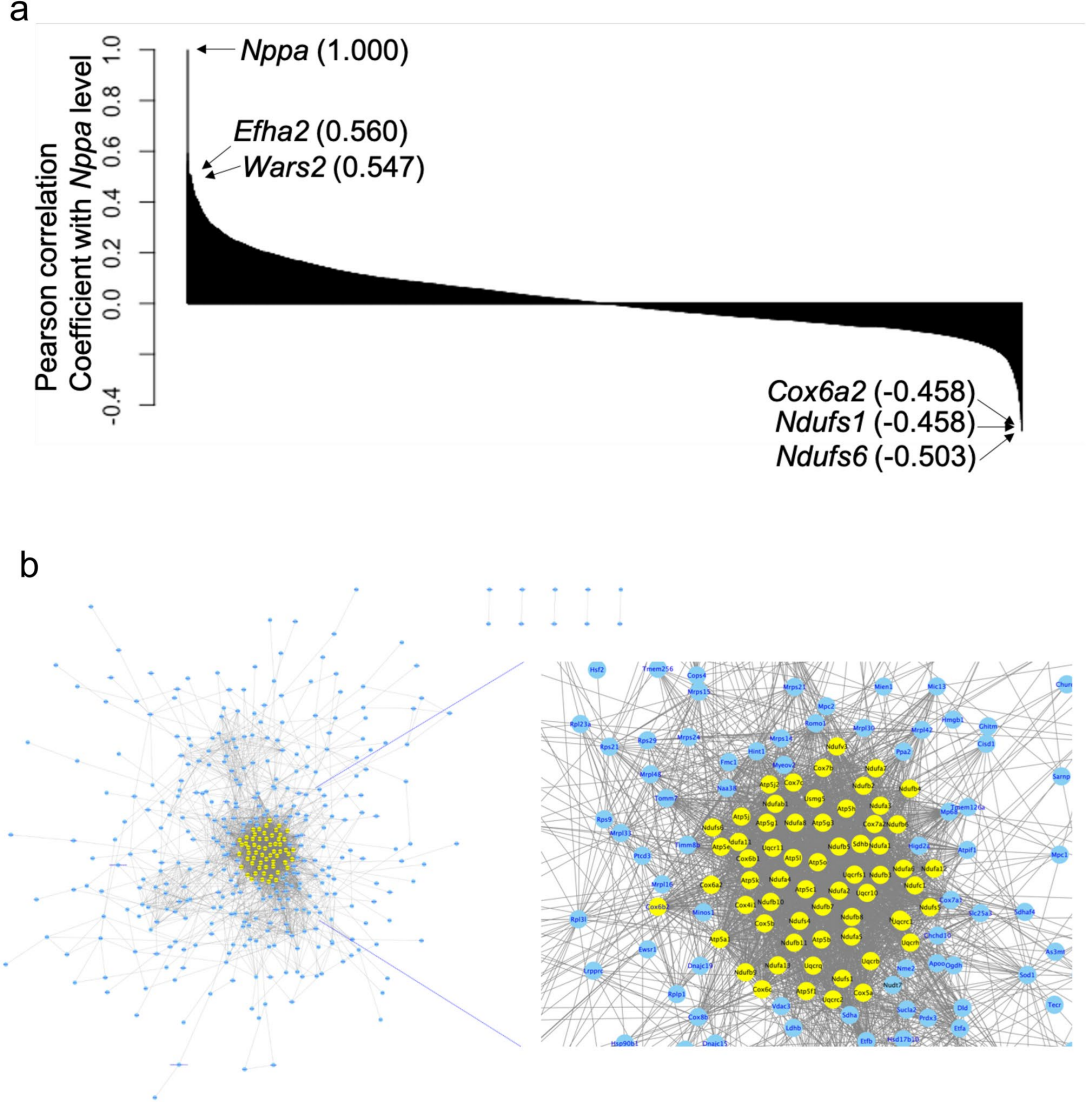
Detection of genes correlated with *Nppa* and visualization of gene–gene association network involving genes negatively correlated with *Nppa*. (**a**) Correlation coefficient between the expression levels of *Nppa* and those of other genes at day 3 after TAC in cardiomyocytes. (**b**) The network shows a graphical representation of gene–gene associations involving 500 negatively correlated genes, where circles indicate genes. Genes involved in oxidative phosphorylation are represented by yellow nodes.

To detect the genes correlated with *Nppa* upregulation in the initial stage of heart failure, we calculated Pearson’s correlation coefficients of expression of *Nppa* and other genes at day 3 after TAC. Figure 7a shows the list of genes whose expression was positively and negatively correlated with that of *Nppa*. The genes positively correlated with *Nppa* are considered upregulated in *Nppa* high-expressing cardiomyocytes, while the genes negatively correlated with *Nppa* are considered downregulated.

Next, we performed GO enrichment analysis on the positively and negatively correlated genes in order to clarify the alteration of biological function in cardiomyocytes with high *Nppa* expression after TAC. Table 1a shows a list of the top 10 GO terms for the genes that were positively correlated with *Nppa*. The corresponding GO terms included ‘microtubule-based process’ and ‘actin cytoskeleton organization’, which are associated with the composition of cellular organelles in the sarcomere system that characterize the function of cardiomyocytes and muscle. Table 1b shows a list of the top 10 GO terms for the genes that were negatively correlated with *Nppa*. The corresponding GO terms included ‘ATP biosynthetic process’, ‘ATP metabolic process’ and ‘oxidation–reduction process’, which are associated with mitochondrial function. These results suggest that, in cardiomyocytes with high *Nppa* expression, the composition of cellular organelles in the sarcomere system that characterize the function of cardiomyocytes and muscle are upregulated and mitochondrial function is downregulated.

**Table 1:** GO terms of genes correlated with *Nppa.* (**a**) List of GO terms of the genes whose expression patterns were positively correlated with that of *Nppa*. (**b**) List of GO terms of the genes whose expression patterns were negatively correlated with that of *Nppa*.

(a) Positively correlated genes		
Category	Term	p-value
G0:0007017	microtubule-based process	6.76E−06
G0:0016310	phosphorylation	1.04E−05
G0:0030036	actin cytoskeleton organization	3.85E−05
G0:0007049	cell cycle	4.78E−05
G0:0001974	blood vessel remodeling	7.22E−05
G0:0007067	mitotic nuclear division	1.65E−04
G0:0098609	cell–cell adhesion	1.90E−04
G0:0016569	covalent chromatin modification	5.16E−04
G0:0006468	protein phosphorylation	6.08E−04
G0:0061025	membrane fusion	6.17E−04

(b) Negatively correlated genes		
Category l	Ferm	p-value
G0:0055114 p	xidation-reduction process	3.48E−30
G0:0006810	ransport	2.66E−11
G0:0015986	TP synthesis coupled proton transport	1.05E−09
G0:0006099	ricarboxylic acid cycle	1.44E−09
G0:0006754	TP biosynthetic process	3.06E−07
G0:0046034	TP metabolic process	8.99E−07
G0:0015992	proton transport	1.21E−06
G0:0008152	metabolic process	4.46E−06
G0:0006635	atty acid beta-oxidation	1.81E−05
G0:0006979	response to oxidative stress	2.51E−05

Mitochondrial dysfunction is considered to be one of the principal mechanisms of heart failure via declining heart function caused by oxidative stress and reduction of ATP synthesis^18,19^. In fact, multiple GO terms correlated with *Nppa* were associated with mitochondrial function, such as ‘oxidation–reduction process’ and ‘ATP biosynthetic process’ in this study. This suggests that mitochondrial dysfunction in cardiomyocytes with high *Nppa* expression may be involved in the pathophysiology of heart failure.

From the viewpoint of systems biology, we attempted to elucidate the overall biological systems by visualizing gene–gene association involving negatively correlated genes in cardiomyocytes with high *Nppa* expression. We then investigated whether the downregulation of mitochondrial function plays a significant role in downregulated biological functions in cardiomyocytes with high *Nppa* expression. We constructed a gene–gene association network using the Search Tool for the Retrieval of Interacting Genes/Proteins (STRING)^20^ based on negatively correlated genes with *Nppa*, and detected modular networks using MCODE^21^ (see the Methods section for more details).

Figure 7b shows the resulting gene–gene association network involving genes whose expressions were negatively correlated with *Nppa*, where the detected modules consisting of 61 genes are colored in yellow. The detected gene modules were involved in the oxidative phosphorylation pathway, and multiple members of the NADH ubiquinone complex family were densely located at the centre of the gene–gene association network. The results suggest that mitochondrial dysfunction plays a central role in the functions of cardiomyocytes with high *Nppa* expression and the related genes work in an interactive manner.

### Nkx2-5 and Gtf2b are predicted to be transcription factors regulating genes characteristic of cardiomyocytes with high *Nppa* expression

We further investigated the molecular mechanisms that regulate gene expression in cardiomyocytes with differential *Nppa* expression by identifying transcription factors (TFs) acting as master regulators of altered cardiomyocyte gene expression. We searched for TFs that were significantly associated with genes whose expression was correlated with *Nppa* based on large-scale ChIP-seq data acquired by ChIP-Atlas^22^ (see the “Methods” section for more details). Statistical significance was evaluated by two-tailed Fisher’s exact probability test (see he “Methods” section for more details).

**Figure 8:**
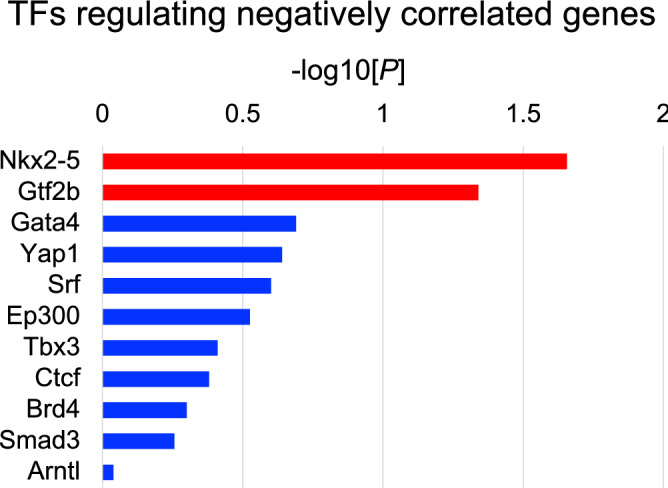
Prediction of transcription factors that regulate genes correlated with *Nppa*. High scoring transcription factors (TFs) for genes correlated with *Nppa* are shown. The horizontal axis in each panel indicates -log_10_*P*-values. TFs with statistical significance (P < 0.05) are represented by red bars, and TFs with non-statistical significance (P > 0.05) are represented by blue bars.

Figure 8 shows ten high scoring TFs associated with genes negatively correlated with *Nppa*. The enrichment scores for NK-2 transcription factor related, locus 5 (Nkx2-5) and general transcription factor II B (Gtf2b) were statistically significant, thus, Nkx2-5 and Gtf2b were identified as TFs regulating genes whose expression was negatively correlated with *Nppa*. In contrast, TFs regulating positively correlated genes were not detected. These results suggest that Nkx2-5 and Gtf2b are transcription factors characteristic of cardiomyocytes with high expression of *Nppa*.

## Discussion

**Figure 9:**
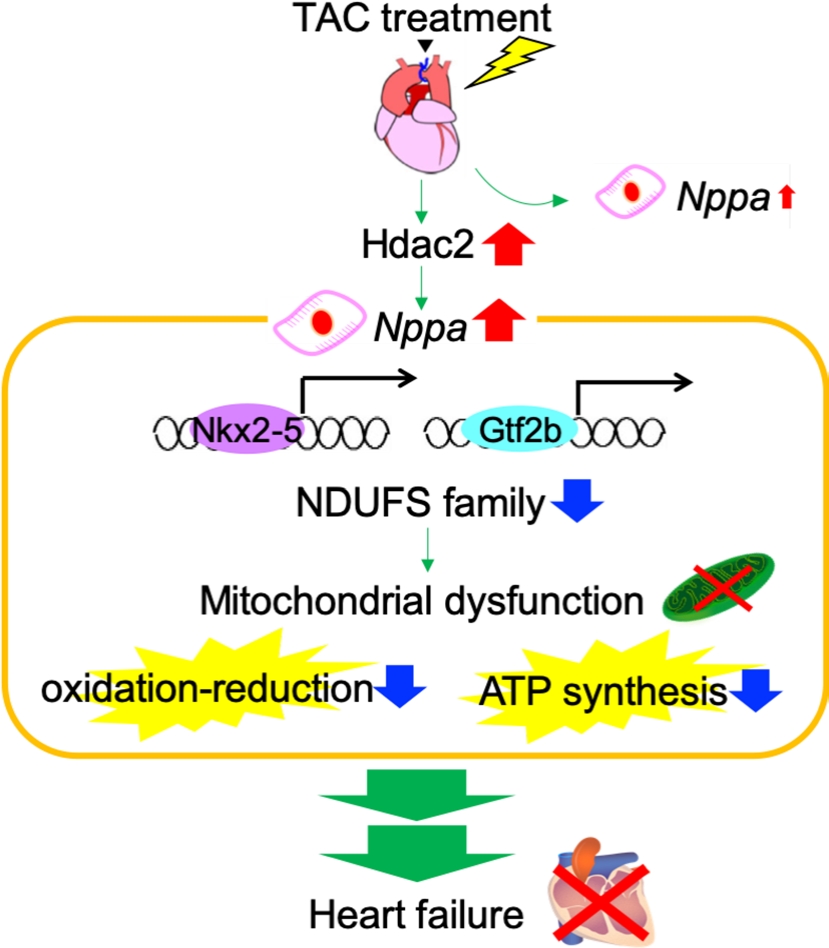
A summary of the molecular mechanisms in the process of heart failure, as inferred by this study. *Nppa* expression level was notably induced by Hdac2 after stress loading, and exhibited cell-to-cell heterogeneity. Under the regulation of Nkx2-5 and Gtf2b, activation of components of muscle and mitochondrial dysfunction were induced, resulting in heart failure.

In this study, we investigated the molecular signatures of heart failure in response to TAC using single-cardiomyocyte transcriptome data. By focusing on the significant induction of *Nppa* in single cardiomyocytes after stress loading, we identified potential molecular mechanisms behind the process of heart failure. *Nppa* expression level was notably induced by Hdac2 after stress loading, and exhibited cell-to-cell heterogeneity. We proposed that under the regulation of Nkx2-5 and Gtf2b, activation of components of muscle and mitochondrial dysfunction were induced in cardiomyocytes with high *Nppa* expression, resulting in heart failure by a trans-omics approach. Figure 9 shows an illustration of the mechanisms estimated by this study.

The novelty of this study is the observation that the expression levels of *Nppa* were variable among cardiomyocytes after TAC and that the upregulation of *Nppa* was observed in a subset of cardiomyocytes (20–30%)*. Nppa,* identified in this study, is a gene encoding atrial natriuretic peptide (ANP). ANP is a cardiac hormone that regulates salt–water balance and blood pressure by promoting renal sodium and water excretion and stimulating vasodilation^23,24^. The *Nppa* gene is expressed primarily in the heart, where its expression level is higher in the atria than in the ventricles^23,25^. In addition, *Nppa*, which has been reported as a stress response marker^23–25^, was shown to be induced in pathological conditions such as cardiac hypertrophy^23–25^, dilated cardiomyopathy^26^ and heart failure^23–25^. Previous studies also reported that *Nppa* expression is regulated by transcription factors such as GATA4^27,28^, Klf4^17,28^ and Hdac2^29^ in response to stress. Klf4 is a novel anti-hypertrophic transcriptional regulator and mediates the HDAC inhibitor-induced prevention of cardiac hypertrophy and *Nppa* upregulation^17,30,31^. However, regulatory mechanisms of *Nppa* induction at the single-cell level remain unknown. In this study, the expression level of Hdac2 was upregulated compared to sham group (Fig. 5). These results suggest that Hdac2 activation is induced after TAC, resulting in the repression of Klf4 transcriptional response to stress and continuous *Nppa* expression in the process of heart failure at the single-cell level.

This study clarified that *Nppa* expression was induced by TAC in a heterogeneous manner among single cardiomyocytes (Fig. 2a,b). The expression of myosin heavy chain β (*Myh7*), a representative fetal gene that is activated in response to hemodynamic overload through cardiac hypertrophy^32,33^, was previously reported to be induced after TAC in a heterogeneous manner among single cardiomyocytes^7^. *Myh7*-expressing cardiomyocytes were significantly more abundant in the middle layer of the heart, than in the inner or outer layer of the heart, at 2 weeks after TAC, while such spatial differences were not observed at 8 weeks after TAC. *Myh7* expression were highly correlated with *Nppa* in cardiomyocyte^7^, suggesting that *Nppa*-expressing cardiomyocytes were more abundant in the middle layer of the heart than in the inner or outer layer of the heart. *Nppa* is also considered as a fetal gene^34^ and demonstrated heterogenous expression among cardiomyocytes. To clarify the molecular mechanism of heterogeneity of *Nppa* expression, we should investigate the correlation between the expression level of *Nppa* and spatial location of cardiomyocytes.

This study also found that cardiomyocytes with high *Npp*a expression demonstrated mitochondrial dysfunction associated with downregulation of NADH ubiquinone complex family (Fig. 6). Mitochondrial dysfunction is one of the principal mechanisms of heart failure because the heart is highly dependent on mitochondrial ATP production and the myocardium possesses the largest number of mitochondria of any tissue^18,19^. Hdac class I activation contributes to mitochondrial dysfunction in cardiomyocytes via regulating tumour necrosis factor-α (TNF-α), which impairs myocardial function by a variety of molecular mechanisms such as the production of reactive oxygen species (ROS) which induces mitochondrial damage^35^. Mitochondrial dysfunction of the heart is correlated with the severity and prognosis of heart failure^18^ suggesting that the measurement of biomarkers that evaluate mitochondrial dysfunction can be used to assess the progression and prognosis of heart failure. This study suggested that the evaluation of *Nppa* expression level from biopsy specimens may be useful to predict the prognosis of patients with heart failure. Our results suggest that *Nppa* upregulation correlates with mitochondrial dysfunction, but it is difficult to judge whether the relationship is direct or not. Fetal genes are downregulated during cardiac development, while they are upregulated in response to hemodynamic overload through cardiac hypertrophy and the progression of heart disease. It is known that the cardiomyocyte lose their functions and become immature during disease progression, and fetal genes play a critical role in the pathogenesis of heart failure. Mitochondrial dysfunction is one of the factors that characterize the metabolic failure of cardiomyocytes (i.e., myocardial immaturity). Based on these findings, *Nppa* upregulation and mitochondrial dysfunction are common indicators of "myocardial immaturity". This implies that *Nppa* up-regulation and mitochondrial dysfunction are biologically related. We would like to investigate the details of these biological findings in our future work.

Two famous biomarkers of heart failure, ANP and brain/B-type natriuretic peptide (BNP), are primarily produced by, and secreted from, heart tissue. Since plasma ANP and BNP concentrations, as well as expression, are elevated in response to increased body fluid volume and pressure load on the heart wall, these peptides are widely utilized as diagnostic biomarkers for evaluating heart failure^23,24,36^. However, the validity of the measurement of *Nppa* and *Nppb* expression level in biopsy specimens has not been verifyed. This study supposed that *Nppb* cannot explain the cell heterogeneity and implies that *Nppa* expression in cardiomyocytes can explain the heterogeneity of cardiomyocytes. We evaluated the expression level of *NPPA* histologically by using human myocardium tissue (Supplementary Fig. 2). The ISH results show that the expression of *NPPA* was significantly upregulated with heterogeneity in the heart of patients with heart failure in comparison with healthy subjects. As further evidence, Sergeeva and Christoffels, in a previous study of the expression of *Nppa* under stress loading in the heart, subjected the hypertrophied hearts of mice to immunostaining^37^. These results demonstrated that *Nppa* was upregulated with heterogeneity in cardiac hypertrophy in mice. The results show that the expression of *NPPA* was significantly upregulated with heterogeneity in the heart of patients with heart failure in comparison with healthy subjects. Combining both the measurement of ANP or BNP blood concentration and the measurement of biopsy *Nppa* expression levels, might improve the accuracy of predicting the pathological progression and prognosis of heart failure.

This study also revealed that genes correlated with *Nppa* were regulated by Gtf2b and Nkx2-5. Gtf2b, is involved in the formation of the RNA polymerase II preinitiation complex and aids in stimulating transcription initiation. It is therefore associated with transcription in the heart which is targeted by microRNAs in pressure-induced cardiac hypertrophy^38^. The homeodomain factor Nkx2-5 is a central regulator of cardiogenesis that specifies the spatial definition, formation, and maintenance of heart structures^39^. Deletion or mutation of Nkx2-5 results in pathological phenotypes such as congenital heart failure and cardiomyopathy^40–42^. This study showed that these TFs are associated with pathological responses to TAC at the single-cell level, suggesting that, Gtf2b regulates global transcription initiation and Nkx2-5 is associated with pathological phenotypes in cardiomyocytes with high *Nppa* expression. Additional analysis is needed to determine whether mitochondrial dysfunction is regulated by Nkx2-5 or Gtf2b in cardiomyocytes with high *Nppa* expression. To investigate the validity, we performed additional analyses on Nkx2-5 using the ChIP-seq data (GSM862698) that contained the sequence results of Nkx2-5 binding regions in the heart of *Mus musculus*. We were able to confirm that Nkx2-5 bound the promoter regions of the genes (e.g., *Ndufa4*, *Ndufab1*and *Ndufa3*) in the gene–gene association network module (as shown in Supplementary Fig. 3). These results suggest the validity of the master regulator.

In conclusion, this study showed that it is possible to identify an initial marker that aids our understanding of the molecular mechanisms involved in heart failure by statistical analysis of time-course data without using pseudo-time analysis. Pseudo-time analysis increases the temporal resolution of transcriptome dynamics collected at multiple pseudo-time points, and can be used to recover single-cell gene expression kinetics from a wide array of cellular processes, including differentiation, proliferation and oncogenic transformation^43^. As a result, it is possible to elucidate the cell dynamics as checkpoints and novel molecular mechanisms related to cell and organ development, cell fate, and disease progression^6,44,45^. However, if single-cell RNA-seq data are observed over time, the information regarding true time axis lost, by using the pseudo-time analysis. The use of the original time points of single cell RNA-seq data could provide the information that pseudo-time analysis fails to extract. Moreover, the trans-omics analysis of single-cell transcriptome data with regulome data can help to understand the detailed molecular mechanisms of disease progression in heart failure. The proposed approach proposed in this study is therefore expected to be useful for the investigation of many other diseases.

## Methods

### Animal model and isolation of cardiomyocytes

This study was carried out in compliance with the ARRIVE guidelines. All animal experiments were approved by the Ethics Committee for Animal Experiments of the University of Tokyo (RAC150001) and Osaka University (22-056) and adhered strictly to the animal experiment guidelines as previously descrived^6^. C57BL/6 mice were purchased from CLEA JAPAN. In brief, 8-week-old mice underwent TAC to induce heart failure or were subjected to a sham operation. Sham-operated mice, which were used as controls, underwent a similar surgical procedure without TAC 2 weeks previously. Cardiomyocytes were isolated from the left ventricular free wall after sham operation and at 3 days and 1, 2, 4 and 8 weeks after TAC using the Langendorff method^6^. To evaluate the early, middle, and late stages of disease progression, we acquired single cardiomyocytes at 3 days and 1, 2, 4, and 8 weeks after TAC. TAC reduces the volume of beats from the aorta, thereby increasing pressure in the left ventricle. It induces cardiac hypertrophy at approximately 2 weeks postoperatively and heart failure at 4–8 weeks in 8-week-old C57bL/6 mice^7,46^. Thus, we chose 3 days and 1,2,4, and 8 weeks for the analysis. We collected the cardiac function data from the mice who underwent the TAC model, where the fractional shortening and left ventricular diastolic diameter were measured in the previous work^6^. Mice whose hearts were not appropriately exposed to pressure overload were excluded from the single-cell RNA-seq analysis.

Enzymatic dissociation using Langendorff perfusion was performed with 37 °C pre-warmed 35 mL enzyme solution (collagenase Type II 1 mg/mL, protease type XIV 0.05 mg/mL, NaCl 130 mM, KCl 5.4 mM, MgCl_2_ 0.5 mM, NaH2PO4 0.33 mM, D-glucose 22 mM, HEPES 25 mM, pH 7.4) at a rate of 3 mL/min. Enzymes were neutralized with fetal bovine serum (FBS) at a final concentration of 0.2%. Cell suspensions were filtered through a 100-μm nylon mesh cell strainer and centrifuged at 100 *g* for 2 min. The supernatant was discarded. Cardiomyocytes were purified from non-cardiomyocytes by discarding the supernatant. Live cardiomyocytes were isolated from precipitated cells containing non-cardiomyocytes by visual selection. Rod-shaped live cardiomyocytes (viability of cardiomyocytes at all the time points, ≥ 80%) were manually collected with a 0.2- to 2-µL pipette, visualized by an inverted microscope (OLYMPUS CKX31) and incubated in Smart-seq2 lysis buffer.

### Single-cell RNA-seq analysis of mouse cardiomyocytes

Subsequent reverse transcription, PCR amplification, and PCR purification were conducted in accordance with the Smart-seq2 protocols as previously described^6^. The efficiency of cDNA library generation was assessed by examining the cycle threshold (Ct) values of the control genes (*Tnnt2*, *Cox6a2*) from quantitative real-time polymerase chain reaction (qRT-PCR) using a CFX96 real-time PCR detection system (Bio-Rad) and by examining the distribution of the lengths of cDNA fragments using a LabChip GX (Perkin Elmer) and/or TapeStation 2200 (Agilent Technologies). The following primer sets were used for qRT-PCR: *Tnnt2* mRNA forward, TCCTGGCAGA GAGGAGGAAG; *Tnnt2* mRNA reverse, TGCAGGTCGA ACTTCTCAGC; *Cox6a2* mRNA forward, CGTAGCCCTC TGCTCCCTTA; and *Cox6a2* mRNA reverse, GGATGCGGAGGTGGTGATAC. A Ct value of 25 was set as the threshold. According to the Smart-seq2 protocol, the remaining cDNA libraries were used for the generation of sequencing libraries, which were subsequently subjected to paired-end 51-bp RNA sequencing on a HiSeq 2500 in rapid mode.

We used RPKM normalization for quantitative gene expression analysis of scRNA-seq data in this study. The RefSeq transcripts (coding and non-coding) were downloaded from the UCSC genome browser (http://genome.ucsc.edu). Using Bowtie (version 1.1.1) with the parameters “-S -m 1 -l 36 -n 2 mm9”, we mapped the readings to the mouse genome (mm9). Using DEGseq (version 1.8.0), RPKM was calculated with reads mapped to the nuclear genome. These procedures of calculating RPKM were also described in a previous study^6^. UMI is known to be another normalization method, and Smart-seq2 was not compatible with UMI. However, the present study applied RNA spike-in to Smart-seq2 and confirmed a good correlation between RNA spike-in concentrations and their expected RPKM values (Supplementary Fig. 1c in Nomura et al. *Nat Commun*. 2018). t-SNE analysis of single cardiomyocyte transcriptome for normal C57BL/6 mice (RPKM values) was performed in two different batches, and it was confirmed that cardiomyocytes could not be classified by batch (Supplementary Fig. 1d in Nomura et al. *Nat Commun*. 2018). On the basis of these observations, we used RPKM normalization for quantitative gene expression analysis. The gene expression profiles with normalized RPKM values were used for hierarchical clustering of cardiomyocyte groups (Fig. 5a), calculation of gene expression levels (Figs. 2b, 3, 5b, 6), and detection of genes correlated with *Nppa* (Fig. 7). We used 22,135 genes that were annotated in Refseq and calculated the expression values in scRNA-seq. We sequenced 482 cells for scRNA-seq. Out of them, 396 cells with more than 5,000 expressed genes (RPKM > 0.1) were used for the data analysis.

Single-cell RNA-seq datasets were acquired from GEO-NCBI (GEO accession number: GSE95143). The numbers of RNA-seq cardiomyocyte samples in the sham group and the TAC group at day 3 and week 1, 2, 4 and 8 after operation were 88, 69, 83, 82, 73 and 87, respectively.

### Gene ontology enrichment analysis for DEGs

The Database for Annotation, Visualization and Integrated Discovery (DAVID)^47^ (https://david.ncifcrf.gov/) was used for GO analysis of 1000 genes whose expression was positively or negatively correlated with *Nppa*. The top three GO terms in the annotation clusters that ranked in the Functional Annotation Clustering function with statistical significance (*P* < 0.05) were extracted. The enrichment *P*-values of all extracted GO terms for each module were calculated using DAVID.

Gene set enrichment analysis (GSEA)^48^ was used to determine whether a priori defined sets of genes showed significantly enriched GO terms. GSEA was also used to identify the GO terms associated with significantly enriched genes in cardiomyocytes with high *Nppa* expression compared with cardiomyocytes with low *Nppa* expression.

### Visualization of gene–gene association network and module detection

A gene–gene association network was constructed from molecular interaction data stored in Search Tool for the Retrieval of Interacting Genes (STRING) Database (https://www.string-db.org/), where gene–gene associations are based on evidence such as experiments, databases, co-expression, neighbourhood, gene fusion, and co-occurrence^21^. We used a dataset of gene–gene associations of *Mus Musculus* in the STRING database. The dataset consisted of 11,944,806 protein–protein interactions involving 21,291 proteins. We extracted the gene–gene association network involving 500 genes that negatively correlated with *Nppa.* The gene–gene association network was visualized by Cytoscape^49^. To detect modular networks, the gene–gene association network was subjected to a graphical theoretical clustering algorithm, Molecular Complex Detection (MCODE)^21^, and modular networks with tightly connected nodes were detected. In this study, MCODE was performed with the following criteria: degree cut-off = 2, node score cut-off = 0.2, k-core = 2 and max. depth = 100.

### Prediction of the associated transcription factors

TFs regulating genes whose expression were correlated with *Nppa* were predicted based on large-scale ChIP-seq data in the ChIP-Atlas database (http://chip-atlas.org/) ^22^. ChIP-Atlas contains 76,217 experimental ChIP-seq and DNase-seq datasets, and the enrichment analysis option enables us to search for proteins such as TFs enriched at given genes and genomic regions. In this study, 1000 genes positively or negatively correlated with *Nppa* were subjected to enrichment analysis to predict TFs compared with randomly selected genes. We used the threshold for significance as 100 calculated by peak-caller MACS2 (-10*Log_10_[MACS2 Q-value]) and the distance range from transcription start site (TSS) as 5000 bp up- or downstream. The locations of TSSs and gene symbols were obtained from refFlat files (at UCSC FTP site: https://genome.ucsc.edu/goldenPath/help/ftp.html). ^50^ Only protein-coding genes were used for this analysis. On the basis of information in the refFlat files, the promoter region was set to “ ± 5,000 bp from the TSS” according to the procedure in the ChIP-Atras database^22^. *P*-values were calculated with two-tailed Fisher’s exact probability test as follows:

$$p=\frac{\left(a+b\right)!\left(c+d\right)!\left(a+c\right)!\left(b+d\right)!}{n!a!b!c!d!}$$

where n is total genes, a is the number of TFs binding heart-specific genes, b is the number of TFs not binding heart-specific genes, c is the number of TFs binding randomly selected genes, and d is the number of TFs not binding randomly selected genes.

### Statistical analyses in the trans-omics approach

Genes whose expression changed across the time points were detected by one-way ANOVA. We applied hierarchical clustering using average linkage algorithm with Euclidean distance and identified expression patterns of genes. We calculated the intracluster sum of squared error (SSE) to identify the number of clusters that was optimal for classifying the genes (Supplementary Fig S3). Supplementary Fig S3a shows the intracluster SSE, and Supplementary Fig S3b shows the rate of decrease in the intracluster SSE when the number of clusters increases by one. A distinct cluster structure is known to be formed when the intracluster SSE is small and its rate of decrease is large^51^. The intracluster SSE decreased as the number of clusters increased. The intracluster SSE was decreased to a greater extent when the numbers of clusters were 2 and 6, but less so after 7. From these results, we decided to classify genes into 6 clusters, and the corresponding threshold value (1.22) was set. To detect DEGs in cardiomyocytes with high and low *Nppa* expression, fold change of gene expression was calculated compared with that in the sham group, using the thresholds of |log_2_Fold Change|≥ 1. Unpaired two-tailed Student’s t-test was applied using adjusted *P* < 0.05. Multiple group comparisons among sham-treated cardiomyocytes and those with high and low *Nppa* expression were performed by one-way ANOVA with Tukey’s post hoc test. All statistical analyses and graphical constructions were performed using R version 3.5.2.

To visualize the distributions of gene expression levels, violin plots were generated with the ‘ggplot2′ package in R. To visualize cell-to-cell variations, dimensional reduction was performed on gene expression profiles using t-SNE algorithm (perplexity = 30) with the ‘Rtsne’ package in R. The location of all points in the map was determined by a stochastic minimization of the Kullback–Leibler divergence of the original distances with respect to the mapped distances.

### RNA in situ hybridization

Human cardiac tissues were fixed with G-Fix (Genostaff), embedded in paraffin on CT-Pro20 (Genostaff), using G-Nox (Genostaff) as a less toxic organic solvent than xylene, and sectioned at 5 µm. RNA in situ hybridization was performed with an ISH Reagent Kit (Genostaff) according to the manufacturer’s instructions as previously descrived^6^. Tissue sections were de-paraffinized with G-Nox and rehydrated through an ethanol series and phosphate-buffered saline (PBS). The sections were fixed with 10% neutral buffered formalin (10% formalin in PBS) for 30 min at 37 °C, washed in distilled water, placed in 0.2 N HCl for 10 min at 37 °C, washed in PBS, treated with 4 µg/mL proteinase K (Wako Pure Chemical Industries) in PBS for 10 min at 37 °C, washed in PBS, and placed in a Coplin jar containing 1 × G-Wash (Genostaff), equal to 1 × saline-sodium citrate. Hybridization was performed with sense and anti-sense probes for the *NPPA* gene (250 ng/mL) in G-Hybo-L (Genostaff) for 16 h at 60 °C. After hybridization, the sections were washed in 1 × G-Wash for 10 min at 60 °C and in 50% formamide in 1 × G-Wash for 10 min at 60 °C. Next, the sections were washed twice in 1 × G-Wash for 10 min at 60 °C, twice in 0.1 × G-Wash for 10 min at 60 °C, and twice in TBST (0.1% Tween 20 in Tris-buffered saline) at room temperature. After treatment with 1 × G-Block (Genostaff) for 15 min at room temperature, the sections were incubated with anti-DIG AP conjugate (Roche Diagnostics) diluted 1:2000 with G-Block (Genostaff; dilated 1/50) in TBST for 1 h at room temperature. The sections were washed twice in TBST and incubated in 100 mM NaCl, 50 mM MgCl2, 0.1% Tween 20, and 100 mM Tris–HCl (pH 9.5). Coloring reactions were performed with NBT/BCIP solution (Sigma-Aldrich) overnight and then washed in PBS. The sections were counterstained with Kernechtrot stain solution (Muto Pure Chemicals) and mounted with G-Mount (Genostaff).

## Supplementary Information

Supplementary Information
