# Supplementary material for: Prediction of single-cell mechanisms for disease progression in hypertrophic remodelling by a trans-omics approach
Source: Sci Rep. 2021 Apr 14;11:8112. doi: 10.1038/s41598-021-86821-y (PMC8047020; doi:10.1038/s41598-021-86821-y)
Supplement: Supplementary file 1 — Supplementary Information [file 41598_2021_86821_MOESM1_ESM.pdf]

Prediction of single-cell mechanisms for disease progression in hypertrophic remodelling by a trans-omics approach

Momoko Hamano[1], Seitaro Nomura[2,3], Midori Iida[1], Issei Komuro[2], and Yoshihiro Yamanishi[1*]

[1]Department of Bioscience and Bioinformatics, Faculty of Computer Science and Systems Engineering, Kyushu Institute of Technology, 680-4 Kawazu, Iizuka, Fukuoka 820-8502, Japan

[2]Department of Cardiovascular Medicine, Graduate School of Medicine, The University of Tokyo, Tokyo 113-8655, Japan

[3]Genome Science Division, Research Center for Advanced Science and Technologies, The University of Tokyo, Tokyo 153-0041, Japan

Supplementary data

Supplementary Figure 1: The proportions of cell types in the form of cardiomyocytes with high or low *Nppa* expression at each time point.

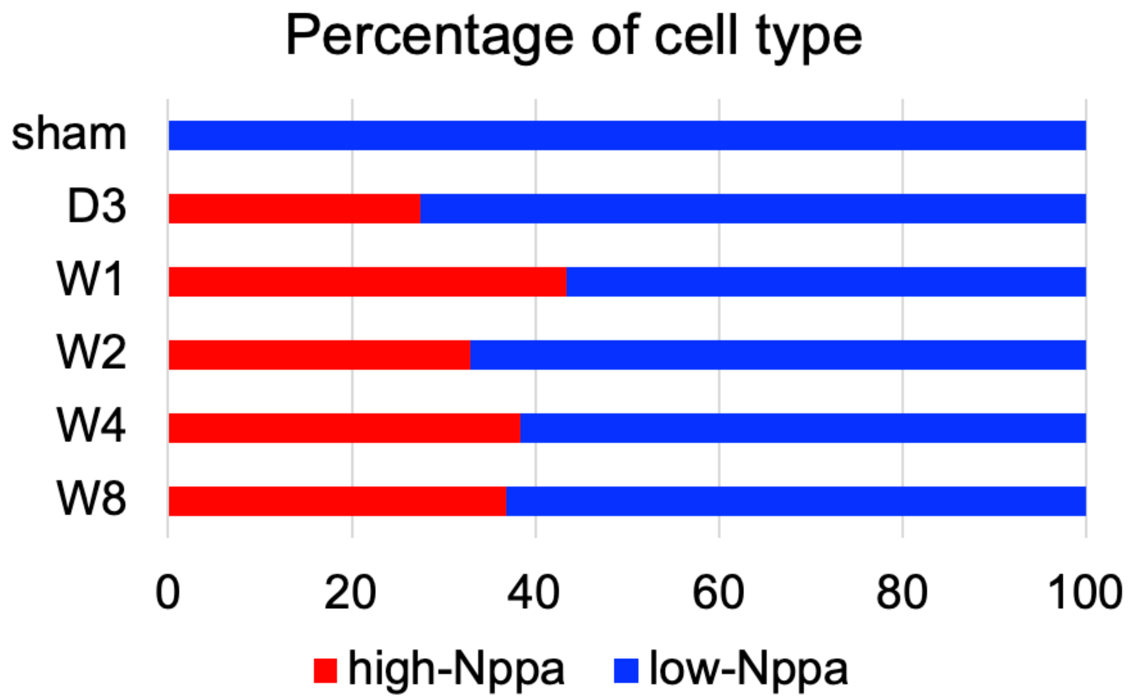

Supplementary Figure 2: Histological evaluation of the expression level of *NPPA* by using human myocardium tissue.

To evaluate the expression distribution of *NPPA* by in situ hybridization (ISH), we used both sense and anti-sense probes for the *NPPA* gene. The scale bar in the figure is 50 µm. The panels in the first row show the expression distribution of *NPPA* mRNA in healthy subjects, and the panels in the third row show the expression distribution of *NPPA* mRNA in patients with heart failure. The panels in the second row show the ISH result using the negative control probe in healthy subjects, and the panels in the fourth row show the result using the negative control probe in patients with heart failure.

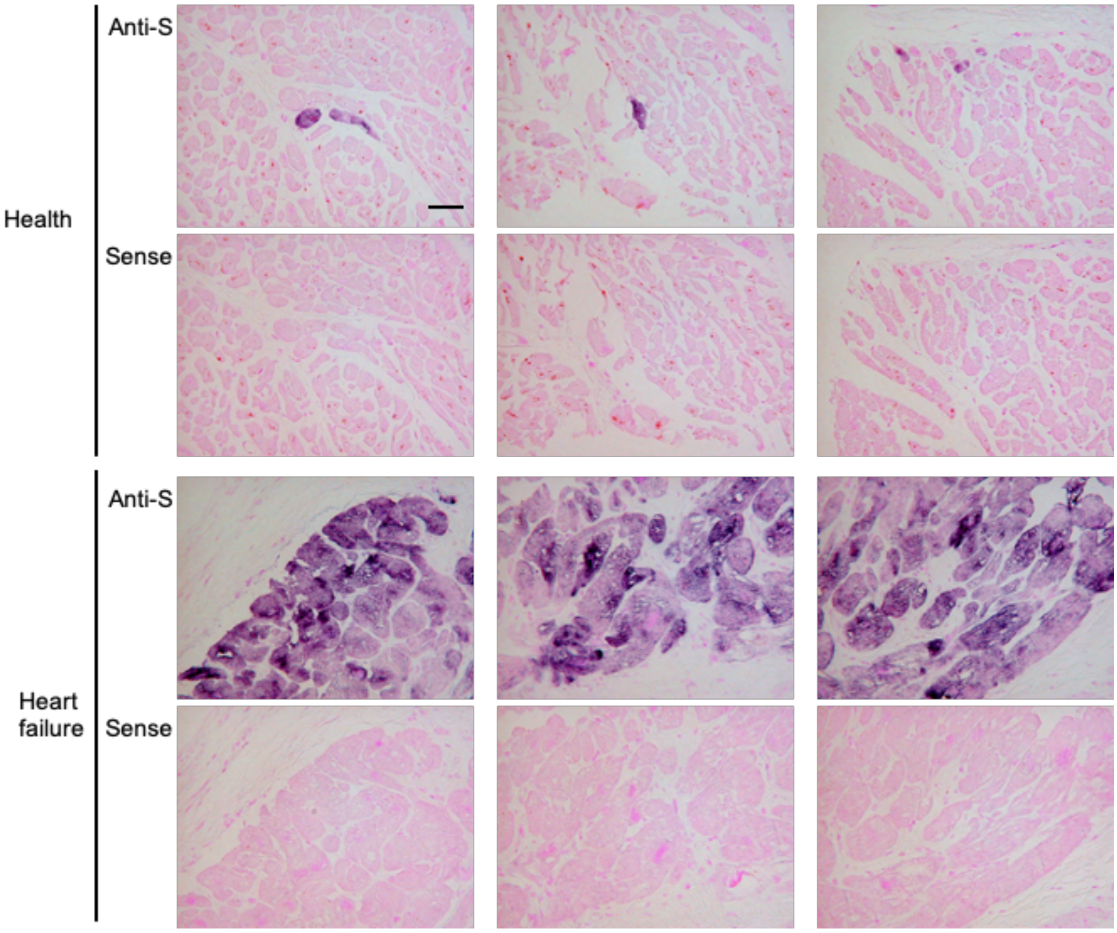

Supplementary Figure 3: Sequence results of Nkx2-5 binding regions in the heart of *Mus musculus*.

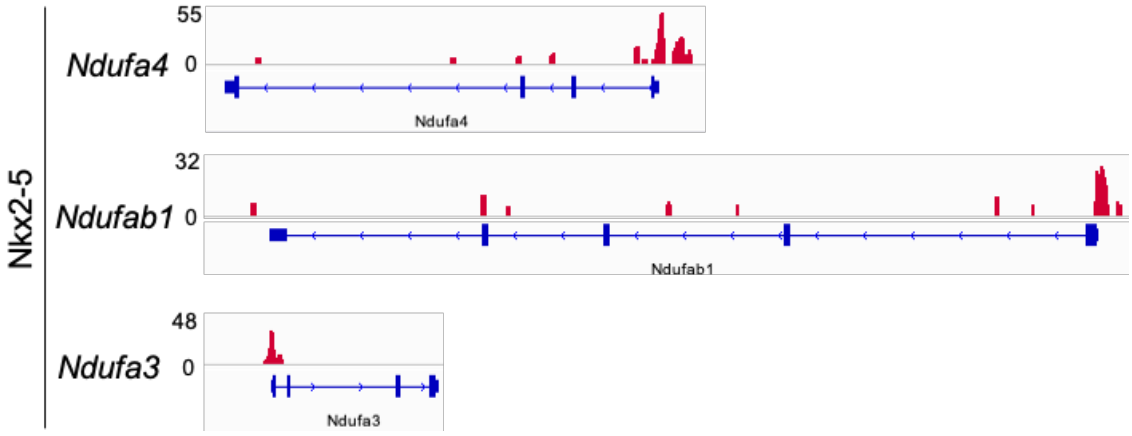

Supplementary Figure 4: Intracluster sum of squared error (SSE) of hierarchical clustering.

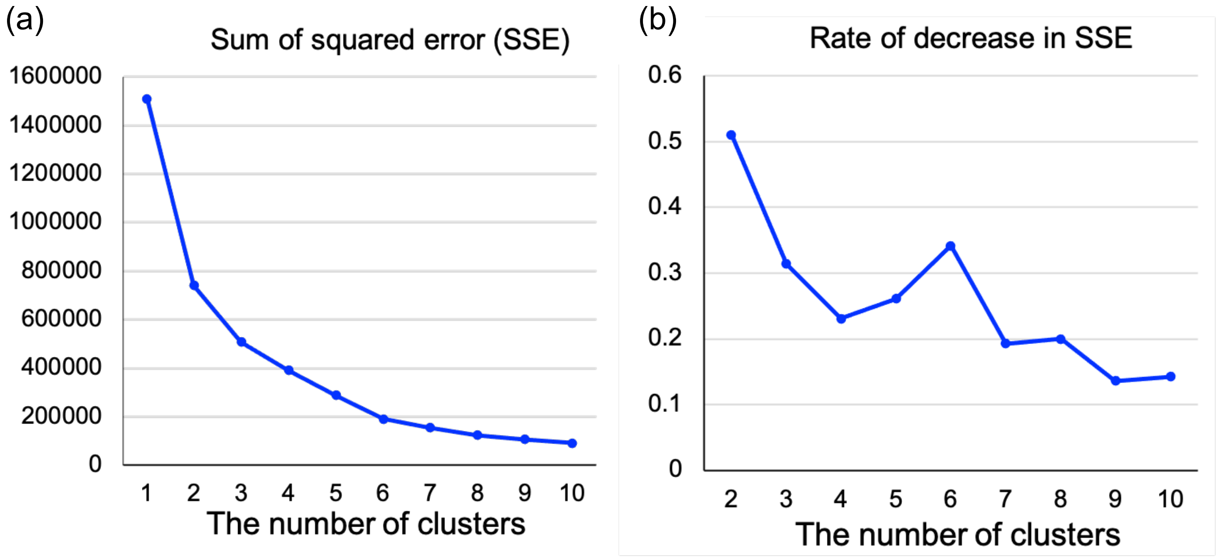

Supplementary Table 1: Top 10 GO terms of the 388 genes in cluster 1 from the 3,135 genes detected by ANOVA.

GO Term	p-value	Fold Enrichment
collagen-activated tyrosine kinase receptor signaling pathway	2.57E-04	29.259
cell-cell adhesion	4.05E-04	3.7154
cell projection organization	0.00114744	3.8754
rRNA processing	0.00143095	4.1798
cell adhesion	0.001724	2.2924
positive regulation of neuron projection development	0.00253866	3.8164
neuron projection development	0.00265484	3.7889
angiogenesis	0.00273804	2.9381
regulation of cell growth	0.00304155	6.0536
protein phosphorylation	0.00475838	2.0319

Supplementary Table 2: Top 10 GO terms of significantly expressed genes in cardiomyocytes with high *Nppa* expression compared with those with low *Nppa* expression at 3 days and 1, 2, 4 and 8 weeks after TAC.

3 days

NAME	NES	NOM p-val
GO_POSITIVE_REGULATION_OF_DENDRITIC_SPINE_DEVELOPMENT	1.7519	0.00000
GO_REGULATION_OF_NATURAL_KILLER_CELL_ACTIVATION	1.7225	0.01058
GO_POSITIVE_REGULATION_OF_AMINE_TRANSPORT	1.6831	0.01443
GO_REGULATION_OF_GRANULOCYTE_CHEMOTAXIS	1.6650	0.00533
GO_NEGATIVE_REGULATION_OF_CYCLIC_NUCLEOTIDE_METABOLIC_PROCESS	1.6614	0.00344
GO_NEGATIVE_REGULATION_OF_BIOMINERAL_TISSUE_DEVELOPMENT	1.6545	0.00528
GO_REGULATION_OF_TRANSCRIPTION_ELONGATION_FROM_RNA_POLYMERASE_II_PROMOTER	1.6477	0.01015
GO_HIPPO_SIGNALING	1.6374	0.00738
GO_DNA_PACKAGING	1.6364	0.00000

1 weeks

NAME	NES	NOM p-val
GO_REGULATION_OF_PHOSPHATIDYLINOSITOL_3_KINASE_ACTIVITY	1.7218	0.00409
GO_REGULATION_OF_METANEPHROS_DEVELOPMENT	1.6886	0.00000
GO_POSITIVE_REGULATION_OF_POTASSIUM_ION_TRANSMEMBRANE_TRANSPORT	1.6583	0.01299
GO_STEROL_TRANSPORT	1.6404	0.00349
GO_REGULATION_OF_POTASSIUM_ION_TRANSMEMBRANE_TRANSPORT	1.6359	0.00383
GO_BILE_ACID_AND_BILE_SALT_TRANSPORT	1.6324	0.00375
GO_DETECTION_OF_MECHANICAL_STIMULUS_INVOLVED_IN_SENSORY_PERCEPTION	1.6312	0.02053
GO_REGULATION_OF_RESPONSE_TO_INTERFERON_GAMMA	1.6221	0.01357
GO_POSITIVE_REGULATION_OF_CALCIUM_ION_TRANSMEMBRANE_TRANSPORTER_ACTIVITY	1.6047	0.01927
GO_ANION_HOMEOSTASIS	1.5985	0.01394

2 weeks

NAME	NES	NOM p-val
GO_NEGATIVE_REGULATION_OF_LYASE_ACTIVITY	1.7194	0.00624
GO_NEGATIVE_REGULATION_OF_CYCLIC_NUCLEOTIDE_METABOLIC_PROCESS	1.7157	0.00714
GO_REGULATION_OF_SYNAPTIC_TRANSMISSION_GLUTAMATERGIC	1.6992	0.00000
GO_REGULATION_OF_GASTRULATION	1.6509	0.00880
GO_PROTEIN_EXIT_FROM_ENDOPLASMIC_RETICULUM	1.5651	0.02194
GO_GLUTAMINE_METABOLIC_PROCESS	1.5606	0.02157
GO_MOTOR_NEURON_AXON_GUIDANCE	1.5481	0.02935
GO_ACTIN_MYOSIN_FILAMENT_SLIDING	1.5480	0.02773
GO_HOMOPHILIC_CELL_ADHESION_VIA_PLASMA_MEMBRANE_ADHESION_MOLECULES	1.5216	0.00000
GO_MAGNESIUM_ION_TRANSMEMBRANE_TRANSPORT	1.5124	0.02030

4 weeks

NAME	NES	NOM p-val
GO_DNA_STRAND_ELONGATION_INVOLVED_IN_DNA_REPLICATION	1.7400	0.00176
GO_GASTRULATION_WITH_MOUTH_FORMING_SECOND	1.7095	0.00171
GO_POSITIVE_REGULATION_OF_PROTEIN_EXPORT_FROM_NUCLEUS	1.6979	0.01207
GO_ZINC_ION_HOMEOSTASIS	1.6921	0.00570
GO_CELL_SUBSTRATE_JUNCTION_ASSEMBLY	1.6880	0.00382
GO_EMBRYONIC_AXIS_SPECIFICATION	1.6590	0.00368
GO_ENTEROENDOCRINE_CELL_DIFFERENTIATION	1.6521	0.00906
GO_REGULATION_OF_SPROUTING_ANGIOGENESIS	1.6472	0.00570
GO_RETINA_LAYER_FORMATION	1.6439	0.00954
GO_ODONTOGENESIS_OF_DENTIN_CONTAINING_TOOTH	1.6388	0.00174

8 weeks

NAME	NES	NOM p-val
GO_EPIBOLY	1.6586	0.00753
GO_SYNAPTIC_TRANSMISSION_GLUTAMATERGIC	1.6246	0.00745
GO_NEGATIVE_REGULATION_OF_LYMPHOCYTE_DIFFERENTIATION	1.6037	0.01601
GO_CRANIAL_NERVE_DEVELOPMENT	1.5976	0.01919
GO_PEPTIDYL_GLUTAMIC_ACID_MODIFICATION	1.5829	0.00874
GO_POSITIVE_REGULATION_OF_ACTIN_CYTOSKELETON_REORGANIZATION	1.5799	0.02348
GO_REGULATION_OF_OSTEOBLAST_PROLIFERATION	1.5540	0.01587
GO_REGULATION_OF_NON_CANONICAL_WNT_SIGNALING_PATHWAY	1.5381	0.01533
GO_REGULATION_OF_MEIOTIC_NUCLEAR_DIVISION	1.5140	0.02564
GO_RESPONSE_TO_PLATELET_DERIVED_GROWTH_FACTOR	1.5133	0.02368
